# A survey of minimally invasive cardiac surgery during the COVID-19 pandemic

**DOI:** 10.1177/02676591211029452

**Published:** 2022-11

**Authors:** Megan Lyons, Enoch Akowuah, Steve Hunter, Massimo Caputo, Gianni D Angelini, Hunaid A Vohra

**Affiliations:** 1 Bristol Medical School, Faculty of Health Sciences, University of Bristol, Bristol, UK; 2 Department of Cardiac Surgery, South Tees Hospital, Middlesborough, UK; 3 Department of Cardiac Surgery, Northern General Hospital, Sheffield, UK; 4 Department of Cardiothoracic Surgery, Blackpool Victoria Hospital, Blackpool, UK; 5 Department of Cardiac Surgery/Cardiovascular Sciences, University of Bristol, Bristol, UK

**Keywords:** MICS, minimally invasive, COVID

## Abstract

**Background:**

Lack of scientific data on the feasibility and safety of minimally invasive cardiac surgery (MICS) during the COVID-19 pandemic has made clinical decision making challenging. This survey aimed to appraise MICS activity in UK cardiac units and establish a consensus amongst front-line MICS surgeons regarding standard best MICS practise during the pandemic.

**Methods:**

An online questionnaire was designed through the ‘googleforms’ platform. Responses were received from 24 out of 28 surgeons approached (85.7%), across 17 cardiac units.

**Results:**

There was a strong consensus against a higher risk of conversion from minimally invasive to full sternotomy (92%; *n* = 22) nor there is increased infection (79%; *n* = 19) or bleeding (96%; *n* = 23) with MICS compared to full sternotomy during the pandemic. The majority of respondents (67%; *n* = 16) felt that it was safe to perform MICS during COVID-19, and that it should not be halted (71%; *n* = 17). London cardiac units experienced a decrease in MICS (60%; *n* = 6), whereas non-London units saw no reduction. All London MICS surgeons wore an FP3 mask compared to 62% (*n* = 8) of non-London MICS surgeons, 23% (*n* = 3) of which only wore a surgical mask. London MICS surgeons felt that routine double gloving should be done (60%; *n* = 6) whereas non-London MICS surgeons held a strong consensus that it should not (92%; *n* = 12).

**Conclusion:**

Whilst more robust evidence on the effect of COVID-19 on MICS is awaited, this survey provides interesting insights for clinical decision-making regarding MICS and aids to facilitate the development of standardised MICS guidelines for an effective response during future pandemics.

## Introduction

Minimally invasive cardiac surgery (MICS) refers to surgical techniques that minimise surgical trauma through smaller incisions^1^ compared to the conventional open sternotomy. MICS has established itself as the standard of care for valve surgery in many cardiac units across the United Kingdom and Europe. This is due to a plethora of benefits, including better cosmesis, fewer blood products, lower risk of infection, quicker recovery and earlier discharge of patients^2,3^; its benefits also span to the high-risk patient groups.^4^ Further, short and long-term survival of patients undergoing MICS is similar to that of conventional approaches when performed in specialist hands.^5^

The World Health Organisation^6^ declared a pandemic on March 11th, 2020 and as of July 11th, there are over 12.5 million confirmed cases of COVID-19.^7^ Cardiac surgery is one of the single largest users of intensive care unit (ICU) beds.^8^ and consequently surgical activity has been significantly affected during this time to facilitate re-allocation of ICU beds, ventilators and staff.^9^ Many cardiac surgeons have also shifted their usual area of expertise to become COVID doctors. The lack of scientific data on the feasibility of MICS practise during the COVID-19 pandemic has made clinical decision making particularly challenging. Questions regarding the safety of MICS have also been raised, particularly during a time of significant staff and resource shortages. This survey aimed to gain an insight into the impact of COVID-19 on MICS and quantitatively appraise MICS activity in cardiac units within the UK during the pandemic. Further, we endeavoured to establish a consensus amongst front-line MICS surgeons regarding best standard MICS practice during this unprecedented time, with particular focus on minimally invasive mitral valve repair (MIMVr) and minimally invasive aortic valve replacement (MIAVR).

## Methods

An online questionnaire survey was designed through ‘googleforms’ platform and sent to 28 leading UK MICS surgeons identified through the British and Irish Society for Minimally Invasive Cardiac Surgery (BISMICS). Responses were received from 24 surgeons (85.7%). Survey responses were submitted and analysed through ‘googleforms’. Participation requests were emailed to the selected surgeons which contained a link to the 34 questions included in the survey. Participation was voluntary and anonymised to the response recipient and the analyst. Questions included in the questionnaire were formulated in an attempt to ascertain the MICS activity in UK cardiac surgical units with particular focus on MICS safety during COVID-19. A strong consensus was pre-defined as an opinion shared by at least 60% of responding consultants. Survey responses were collated and analysed through ‘googleforms’.

## Results

**Figure 1.**
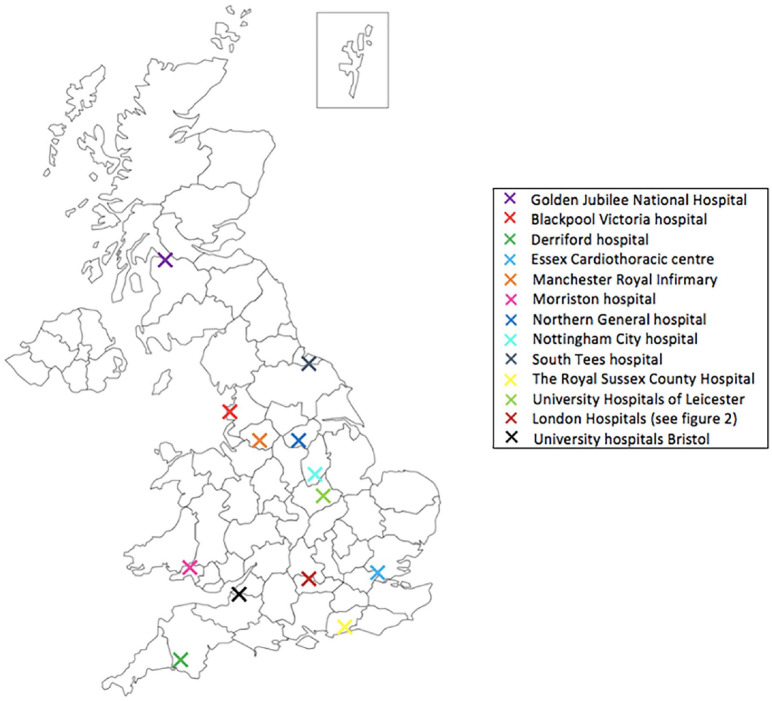
An illustration of the distribution of responding cardiac units: Blackpool Victoria hospital (Blackpool), Derriford Hospital (Plymouth), Essex Cardiothoracic centre (Basildon), Manchester Royal Infirmary (Manchester), Morriston Hospital (Swansea), Northern General Hospital (Sheffield), Nottingham City Hospital (Nottingham), South Tees Hospital (Middlesbrough), The Royal Sussex County Hospital (Brighton), University Hospitals of Leicester (Leicester), University Hospitals Bristol (Bristol), Golden Jubilee National Hospital (Glasgow), London Hospitals.

**Figure 2.**
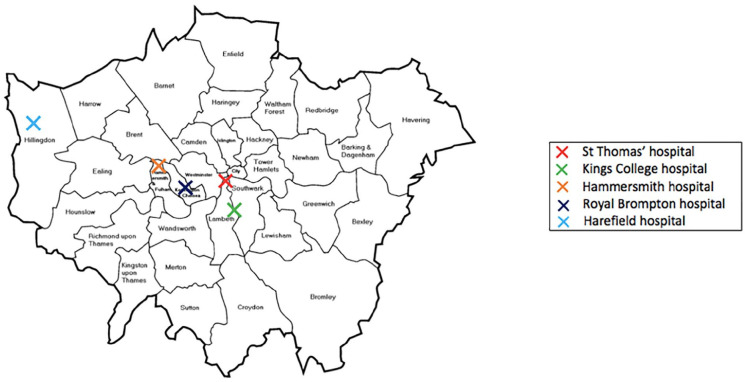
An illustration of the distribution of responding London cardiac units.

**Figure 3.**
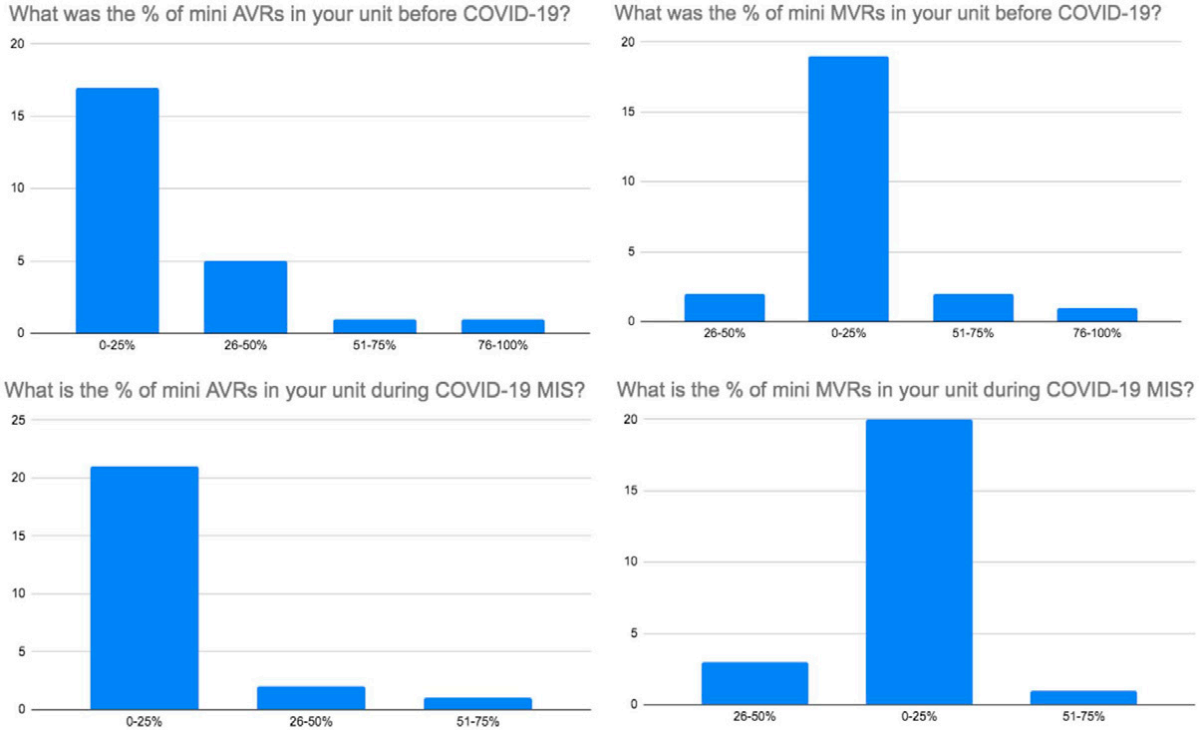
Survey results comparing the change in percentage of mini AVRs (left) and mini MVrs (right) performed before and during COVID-19.

A total of 24 MICS surgeons took part in the survey which enabled us to receive information from 17 UK cardiac units. Geographically, London had the highest number of responding surgeons in the survey. Figures 1 and 2 show the distribution of responding surgeons across London and non-London regions. The survey showed that 58% (*n* = 14) of MICS surgeons who responded in the survey perform both MIMVr and MIAVR as part of their routine clinical practise. It was shown that during COVID-19, MICS was performed in 71% (*n* = 12) of the responding cardiac units. About 33% (*n* = 8) of respondents believed that it was acceptable to perform MICS during the pandemic whilst 46% (*n* = 11) felt that it was acceptable but only in experienced hands. The remaining 21% (*n* = 5) remained undecisive. Based on surgeons’ responses, the survey confirmed that overall there was a decrease in the number of both MIMVr and MIAVR performed during COVID-19 compared to the pre-pandemic era (Figure 3).

**Figure 4.**
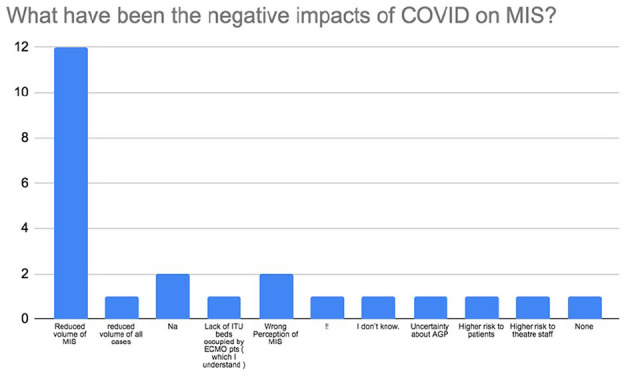
Survey results on the negative impact of COVID-19 on MICS.

**Figure 5.**
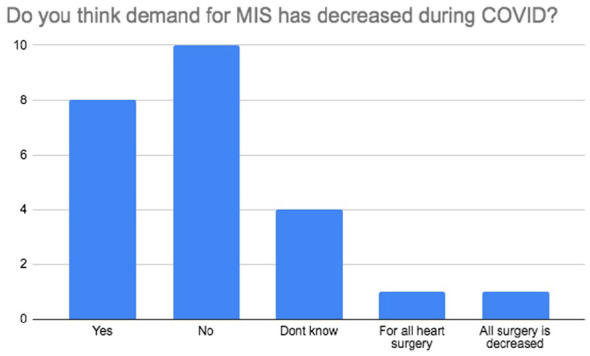
Survey results on the demand for MICS during COVID-19.

There was a strong consensus amongst the surgeons in that there was no higher risk of conversion from MICS to full sternotomy (92%; *n* = 22) nor there was increased infection (79%; *n* = 19) or bleeding (96%; *n* = 23) with MICS compared to full sternotomy during the pandemic. The majority of participants (67%; *n* = 16) felt that it was safe to perform MICS during COVID-19 and that it should not be stopped during this time (71%; *n* = 17). About 63% (*n* = 15) of respondents felt that there was no higher risk of death with MICS during the pandemic but interestingly, 13% (*n* = 3) stated that they believed there was. Over half of respondents believed that COVID-19 has had damaging effects on MICS with 50% (*n* = 12) feeling that the overarching negative impact is due to a reduced volume of MICS being performed (Figure 4). The majority of all participants (79%; *n* = 19) also believed that routine double gloving during the pandemic is not required. There was disparity in responses to the question regarding demand of MICS during the pandemic. While 42% (*n* = 10) of MICS surgeons believed that demand for MICS had not decreased during COVID-19, 33% (*n* = 8) felt that it had (Figure 5). A wide variety of responses were obtained to the open ended question to the MICS surgeons regarding opinion on MICS practice during COVID-19. Table 1 shows these responses separated by London and non-London. Analysis of responses per geographical region showed that 80% (*n* = 8) of responding London surgeons continued to perform MICS during COVID-19. Only 40% (*n* = 4) of London MICS surgeons felt that it was acceptable to do so during the pandemic. This is compared to 69% (*n* = 9) of non-London surgeons performing MICS during the pandemic and 85% (*n* = 11) of non-London MICS surgeons stating that it was acceptable to do so. About 40% (*n* = 4) of London MICS surgeons reported a decrease in the percentage of MIMVr and MIAVR activity during COVID-19, whereas non-London surgeons had seen no reduction in MIMVr or MIAVR. Similar to London, non-London MICS surgeons shared the strong belief that there is not a higher risk of conversion to sternotomy (92%; *n* = 12) or higher risk of infection (85%; *n* = 11) with MICS during the pandemic. However, despite 60% (*n* = 6) London MICS surgeons felt that there was no higher risk of death with MICS during COVID-19, (40%; *n* = 4) compared to 23% (*n* = 3) non-London surgeons believed that was the case.

**Table 1.** Free text responses by specialists regarding opinion on MICS practice during COVID-19.

Non-London responses	London responses
Avoid it.	Massive setback
Should continue – especially in green patients – we are just getting some normality now (July) if PPE relaxed it will be easier for all staff – with mini mitral changing et tube from double to single at end case has caused anxiety – in anaesthesia – longer case so longer in PPE has been difficult for those scrubbed – MIS needs continued support	It has its place in experienced centres with appropriate COVID protection equipment
Do as normal. Select patients well, be meticulous and stick to basics. Patients are better screened anyway.	Experienced surgeons should continue to do them and consent include the COVID effects
Just carry on as normal the length of stay for my patient is 2–5 days so they are discharged earlier	Do it
Continue with routine practise but defer priority three or four cases till local incidence of C19 reduces	Fine as long as done in expert centre
I would support it by any means. In our hospital the MIS was postponed even before C19 outbreak	No change
Added risk for upper airway management for anaesthetic staff	No change in experienced hands
Should be carried out as before for elective patients	
Mini AVRs are less riskier than mini mitrals	
Safe and effective in experienced hands	
Needs to be done as usual	
Carry on as usual	

The majority of all participants (79%; *n* = 19) also believed that routine double gloving during the pandemic is not required; predominantly emanating from non-London MICS surgeons (92%; *n* = 12).

This survey showed that all London MICS surgeons wore FP3 or similar mask during MICS in COVID-19 whereas just 62% (*n* = 8) of non-London MICS surgeons wore FP3 or similar mask and 23% (*n* = 3) wore only a surgical mask throughout the pandemic.

Although the majority of London and non-London MICS surgeons strongly agreed that COVID-19 has had damaging effects on MICS (60%; *n* = 6 and 62%; *n* = 8 respectively), there seemed to be a clear consensus for a favourable approach to continuing MICS surgery amongst non-London surgeons (85%; *n* = 11), yet 60% (*n* = 6) of London surgeons felt that MICS should be halted during the pandemic. A full list of questions and results are shown in Table 2.

**Table 2.** Full list of questions and results; highest percentage answer highlighted.

Question:	Answers (% and *n*)
1. What hospital do you work in?	See Figure 1
2. How many consultant cardiac surgeons work in your unit?	4 (4.2%; *n* = 1)
	5 (20.8%; *n* = 5)
	6 (8.3%; *n* = 2)
	7 (29.2%; *n* = 7)
	8 (25%; *n* = 6)
	9 (4.2%; *n* = 1)
	10 (4.2%; *n* = 1)
	12 (4.2%; *n* = 1)
3. Are you?	Consultant (100%; *n* = 24)
	Registrar (0%)
4. Do you conduct minimally invasive cardiac surgery?	Yes (95.8%; *n* = 23)
	No (4.2%; *n* = 1)
5. What minimally invasive valve surgery do you perform?	Both AVR and MVR (58.3%; *n* = 14)
	Only AVR (37.5%; *n* = 9)
	Only MVR (4.2%; *n* = 1)
6. How many AVR cases do you conduct per year?	0 (4.2%; *n* = 1)
	1–10 (12.5%; *n* = 3)
	11–20 (29.2%; *n* = 7)
	21–30 (29.2%; *n* = 7)
	31–40 (8.3%; *n* = 2)
	40+ (16.7%; *n* = 4)
7. How many mini MV operations do you conduct per year?	0 (33.3%; *n* = 8)
	1–10 (25%; *n* = 6)
	11–20 (12.5%; *n* = 3)
	21–30 (8.3%; *n* = 2)
	31–40 (4.2%; *n* = 1)
	40+ (16.7%; *n* = 4)
8. How many cases of MIDCAB surgery do you conduct per year?	0 (66.7%; *n* = 16)
	1–10 (16.7%; *n* = 4)
	11–20 (0%; *n* = 0)
	21–30 (4.2%; *n* = 1)
	31–40 (0%; *n* = 0)
	40+ (12.5%; *n* = 3)
9. How many cases of minimally invasive atrial fibrillation ablation do you conduct per year?	0 (62.5%; *n* = 15)
	1–10 (20.8%; *n* = 5)
	11–20 (12.5%; *n* = 3)
	21–30 (4.2%; *n* = 1)
	31–40 (0%; *n* = 0)
	40+ (0%; *n* = 0)
10. How do you cannulate for cardio-pulmonary bypass during MIS?	Arterial – Femoral (62.5%; *n* = 15)
	Arterial – Aortic (54.2%; *n* = 13)
	Venous – Femoral (50%; *n* = 12)
	Venous – Right atrial (41.7%; *n* = 10)
	Femoral and Aortic (4.2%; *n* = 1)
	Fem-Fem for Mitral, Fem-Aorta for Aortic (8.4%; *n* = 2)
11. Do you use vacuum-assisted venous drainage for MIS?	Yes (91.7%; *n* = 22)
	No (8.3%; *n* = 2)
12. What cardioplegia do you use during MIS?	Blood: Warm (4.2%; *n* = 1)
	Blood: Cold (83.3%; *n* = 20)
	Crystalloid: Warm (0%; *n* = 0)
	Crystalloid: Cold (4.2%; *n* = 1)
	Warm induction, cold load, intermittent cold and terminal hot shot (4.2%; *n* = 1)
	Custodial first mitral cold blood for mini AVR (4.2%; *n* = 1)
13. Has MIS been performed in your unit during COVID-19?	Yes (70.8%; *n* = 17)
	No (29.2%; *n* = 7)
14. Do you think it is acceptable/a good idea to perform MIS during the pandemic?	Yes (33.3%; *n* = 8)
	No (12.5%; *n* = 3)
	Yes but only in experienced hands (45.8%; *n* = 11)
	Don’t know (4.2%; *n* = 1)
	Only minis I have done are category 2 patients (4.2%; *n* = 1)
15. What was the % of mini AVRs in your unit before COVID-19?	0%–25% (70.8%; *n* = 17)
	26%–50% (20.8%; *n* = 5)
	51%–75% (4.2%; *n* = 1)
	76%–100% (4.2%; *n* = 1)
16. What is the % of mini AVRs in your unit during COVID-19?	0%–25% (87.5%; *n* = 21)
	26%–50% (8.3%; *n* = 2)
	51%–75% (4.2%; *n* = 1)
	76%–100% (0%; *n* = 0)
17. What was the % of mini MVRs in your unit before COVID-19?	0%–25% (79.2%; *n* = 19)
	26%–50% (8.3%; *n* = 2)
	51%–75% (8.3%; *n* = 2)
	76%–100% (4.2%; *n* = 1)
18. What is the percentage of mini MVRs in your unit during COVID-19?	0%–25% (83.3%; *n* = 20)
	26%–50% (12.5%; *n* = 3)
	51%–75% (4.2%; *n* = 1)
	76%–100% (0%; *n* = 0)
19. Do you think there is a higher risk of conversion to sternotomy with MIS during the pandemic?	Yes (0%; *n* = 0)
	No (91.7%; *n* = 22)
	Don’t know (8.3%; *n* = 2)
20. Do you think there is a higher risk of infection with MIS compared to sternotomy during the pandemic?	Yes (4.2%; *n* = 1)
	No (79.2%; *n* = 19)
	Don’t know (12.5%; *n* = 3)
	Possible (4.2%; *n* = 1)
21. Do you think there is a higher risk of coronavirus transmission with MIS during the pandemic?	Yes (20.8%; *n* = 5)
	No (58.3%; *n* = 14)
	Don’t know (12.5%; *n* = 3)
	Consider the impact of CO_2_ in suffocation (4.2%; *n* = 1)
	Possible (4.2%; *n* = 1)
22. Do you think there is a higher risk of bleeding with MIS during the pandemic compared to before the pandemic?	Yes (0%; *n* = 0)
	No (95.8%; *n* = 23)
	Don’t know (4.2%; *n* = 1)
23. Do you think there is a higher risk of death with MIS during the pandemic?	Yes (12.5%; *n* = 3)
	No (62.5%; *n* = 15)
	Don’t know (16.7%; *n* = 4)
	Possible (4.2%; *n* = 1)
	No different to sternotomy (4.2%; *n* = 1)
24. Do you think demand for MIS has decreased during COVID?	Yes (33.3%; *n* = 8)
	No (41.7%; *n* = 10)
	Don’t know (16.7%; *n* = 4)
	For all heart surgery (4.2%; *n* = 1)
	All surgery is decreased (4.2%; *n* = 1)
25. In your opinion, do you think it is safe to perform MIS during COVID?	Yes (66.7%; *n* = 16)
	No (16.7%; *n* = 4)
	Only in experienced hands (8.3%; *n* = 2)
	In a unit with well-established programme yes (4.2%; *n* = 2)
	If the COVID status is negative then it is safe (4.2%; *n* = 1)
26. Do you feel that minimally invasive cardiac surgery should be halted during COVID?	Yes (16.7%; *n* = 4)
	No (70.8%; *n* = 27)
	No MISMVR, AVR yes (8.3%; *n* = 2)
	At the beginning yes (4.2%; *n* = 1)
27. What PPE do you wear during the pandemic for MIS?	Surgical mask only (20.8; *n* = 5)
	FP3 or similar mask (66.6%; *n* = 16)
	Respirator with filters only (4.2%; *n* = 1)
	Powered respirator hood (4.2%; *n* = 1)
	Started with the hood, now FP3 (4.2%; *n* = 1)
28. Which of the following PPE do you think should be worn during the pandemic for MIS?	Surgical mask only (37.5.8; *n* = 9)
	FP3 or similar mask (45.8%; *n* = 11)
	Respirator with filters only (0%; *n* = 0)
	Powered respirator hood (4.2%; *n* = 1)
	Depends on how the surgeon copes with PPE (4.2%; *n* = 1)
	We only treat COVID protected patients (4.2%; *n* = 1)
	If green patient, normal protection (4.2%; *n* = 1)
29. Do you think routine double gloving should be done during the pandemic in MIS?	Yes (20.8; *n* = 5)
	No (79.2; *n* = 19)
30. Do you feel that COVID has had damaging effects on MIS?	Yes (54.2%; *n* = 13)
	No (33.3%; *n* = 8)
	Don’t know (12.5%; *n* = 3)
31. What have been the negative impacts of COVID on MIS?	Reduced volume of MIS (50%; *n* = 12)
	Higher risk to patients (4.2%; *n* = 1)
	Higher risk to theatre staff (4.2%; *n* = 1)
	Na (8.3%; *n* = 2)
	Wrong perception of MIS (8.3; *n* = 2)
	Reduced volume of all cases (4.2%; *n* = 1)
	Lack of ITU beds occupied by ECMO patients (4.2%; *n* = 1)
	!! (4.2%; *n* = 1)
	Don’t know (4.2%; *n* = 1)
	Uncertainty about AGP (4.2%; *n* = 1)
	None (4.2%; *n* = 1)
32. What is your opinion on doing minimally invasive cardiac surgery during COVID?	See Table 1

## Discussion

This is the first survey of its kind that aims to assess the effect of COVID-19 on MICS practice. The pandemic brought a significant burden upon the National Health Service, particularly in London where the initial phase of COVID-19 was felt the most. The survey’s results demonstrated this as we found that only London hospitals had experienced a reduction in MICS throughout this time. A pan-London approach was swiftly established in March within cardiac surgery^10^ to maximise reallocation of resources to the COVID-19 response and maintain provision of urgent and emergency cardiac surgery. Elective cardiac surgery was thus put on hold. Therefore, the main causes of a reduction of the number of elective valve operations during the pandemic may possibly be due to higher reluctancy of patients to undergo elective surgery during the pandemic, as well as a reduction of critical care beds due to their occupancy by COVID patients. Survey results indeed showed that the majority of surgeons believed that MICS should be halted during this time, despite the fact that the majority of cardiac surgeons expressed opinion that demand for MIS had not decreased. The risk of valve-related morbidity and mortality arising from the postponement of MICS has to be delicately balanced with the risk of contracting COVID-19 during admission for MIMVr and MIAVR procedures. There are many unknowns on both sides, making this a large area of uncertainty. The majority of responding MICS surgeons believed that they should continue their practise as normal throughout the pandemic, but questions regarding the safety and feasibility of MICS during this unprecedented time have been raised. Until robust evidence is published, currently it can be presumed that the risk of acquiring COVID-19 during a hospital stay is equal in both MICS and conventional open surgery. However, one could argue that a midline MICS incision reduces aerosolisation and the associated quicker recovery and shorter hospital stay minimises the risk of acquiring nosocomial COVID-19. This also ‘frees up’ more beds to become available for other patients during a time of bed crisis. One may be erroneously led to believe that the mini-thoracotomy approach used in MIMVr and MIAVR may lead to a higher risk of lung injury compared to a midline full sternotomy,^11^ and lead to potential transmission of COVID-19 to members of staff or worsening of lung injury if the patient were to acquire COVID-related pneumonitis. However, there is no evidence for this and is yet to be proven. The data on number of operations on COVID positive patients was not collected during this survey and hence cannot be reported on, however, acute pulmonary dysfunction due to COVID in patients requiring cardiac surgery is a major concern.

The survey demonstrated a reduction in MICS activity during the COVID-19 pandemic. One should not ignore a risk of reduced expertise during this time due to the reduced activity of MICS and reallocation of cardiac surgeons to COVID-related roles in the preceding months. This may result in less favourable outcomes for patients, particularly in less experienced hands, and only adds to the existing areas of uncertainty about the applicability of MICS during the pandemic.

**Figure 6.**
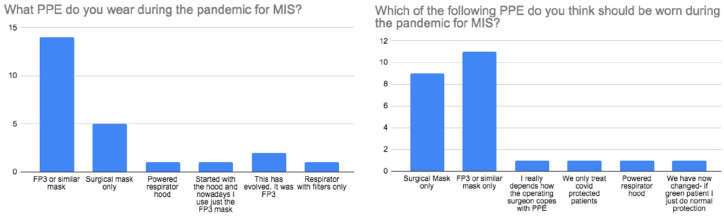
Survey results regarding PPE worn and the opinion of the responders on what PPE should have been worn for MICS during the pandemic.

Based on our survey, we found that although 67% of MICS surgeons wore the FP3 mask (Figure 6), less (46%) believed that FP3 mask should be worn during MICS and felt that a surgical mask would suffice. Although the effects of full PPE on surgeons and theatre staff is difficult to quantify, one would presume that communication, vision, dexterity and concentration would all be significantly impaired, ultimately resulting in worse clinical outcomes. Hence, the tendency to opt for a simple surgical mask in MICS. A trade-off arises between surgical performance and the risk of infection to highly skilled MICS teams, of which there is a finite number nationally. Conversely, the use of reduced PPE could have adverse impacts on urgent and emergency operations if staff shortages mean expertise is not available.

As lockdown measures are eased and hospitals gradually return to a sense of normality, there is a requirement for patient risk stratification. It is now mandatory for patients on elective waiting lists for cardiac surgery to have negative COVID-19 nasopharyngeal swabs and to have completed a 2-week self-isolation period.^12^ The pre-operative CT chest that was once a requirement in the initial COVID-19 phase has now been discarded in the majority of cardiac units due to a lack of evidence of benefit. This only highlights our survey’s limitation of it being just a snapshot of a rapidly evolving era, affecting different regions with variable responses to the pandemic at different times. It is not only wise to make modifications throughout the pandemic but a requisite to ensure that adequate cardiac surgical care can be provided. Further, the issue over whether patient consent should be amended for those who were consented for MICS before COVID-19 arises. Although not best practise to make changes to consent, patients should be made aware of the added risks associated with acquiring COVID-19 during hospital admission and the implications this could have.

The current measures in place for MICS are based upon limited, mostly anecdotal evidence. Thus, guidelines require constant reviewing as evidence-based data is gathered. The applicability of MICS in the midst of a pandemic ultimately remains uncertain and a retrospective systematic analysis based on real world data will be required to understand the clinical impact of COVID-19 on MICS, the findings of which will complement this survey.

## Conclusion

It can be concluded, based on this survey, that MICS was performed in the majority of responding cardiac units within the UK, with only London units experiencing a reduction in MICS activity. Despite this, 67% of respondents believe that MICS is safe which is the opposite recommendation of surgeons from London, who recommend to halt MICS. It is therefore urgent to establish guidelines for the sake of patients. Whilst more robust evidence on the effect of COVID-19 on MICS is awaited, this survey provides some interesting insights for clinical decision-making regarding MICS and aids to facilitate the development of standardised MICS guidelines for an effective response during future pandemics.
